# Supplementary material for: Ipragliflozin Additively Ameliorates Non-Alcoholic Fatty Liver Disease in Patients with Type 2 Diabetes Controlled with Metformin and Pioglitazone: A 24-Week Randomized Controlled Trial
Source: J Clin Med. 2020 Jan 18;9(1):259. doi: 10.3390/jcm9010259 (PMC7019437; doi:10.3390/jcm9010259)
Supplement: Supplementary file 1 [file jcm-09-00259-s001.zip › jcm-692017-SI.doc]

**Figure S1.** Study flow diagram

| **Table S1.** Noninvasive calculation methods for assessing liver steatosis | |
| --- | --- |
| **Models** | **Equations** |
| Fatty liver index | (e ^0.953×loge (triglycerides) + 0.139*BMI + 0.718×loge (GGT) + 0.053×waist circumference - 15.745)^ / (1 + e ^0.953×loge (triglycerides) + 0.139×BMI + 0.718×loge (GGT) + 0.053×waist circumference - 15.745^) × 100 |
| NAFLD liver fat score | - 2.89 + 1.18 × metabolic syndrome (yes = 1/no = 0) + 0.45 × diabetes (yes = 2/no = 0) + 0.15 × (fasting insulin, μU/L) + 0.04 × AST + 0.94 × AST/ALT ratio |
| NAFLD, non-alcoholic fatty liver disease; BMI, body mass index; AST, aspartate aminotransferase; ALT, alanine transaminase; WC, waist circumference; HDL, high-density lipoprotein; GGT, γ-glutamyl transpeptidase. | |
